# Trends of High-Impact Studies in Pharmacology and Pharmacy: A Cross-Sectional Study

**DOI:** 10.3389/fphar.2021.726668

**Published:** 2021-09-16

**Authors:** Lingmin Chen, Yi Yang, Jin Fan, Yonggang Zhang, Nian Li

**Affiliations:** ^1^ Department of Anesthesiology and National Clinical Research Center for Geriatrics, West China Hospital, Sichuan University and The Research Units of West China (2018RU012), Chinese Academy of Medical Sciences, Chengdu, China; ^2^ Department of Clinical Medicine, Gansu University of Traditional Chinese Medicine, Lanzhou, China; ^3^ School of Health Preservation and Rehabilitation, Chengdu University of Traditional Chinese Medicine, Lanzhou, China; ^4^ Chinese Evidence-based Medicine Center, West China Hospital, Sichuan University, Chengdu, China; ^5^ Department of Periodical Press and National Clinical Research Center for Geriatrics, West China Hospital, Sichuan University, Chengdu, China; ^6^ Department of Medical Administration, West China Hospital, Sichuan University, Chengdu, China

**Keywords:** high-impact studies, pharmacology and pharmacy research, visual analysis, cancer, InCites, Web of Science

## Abstract

**Objective:** To investigate the trends of high-impact studies in pharmacology and pharmacy research and to provide evidence for future research in the field of pharmacology and pharmacy.

**Methods:** A cross-sectional study was performed to understand the current status of high-impact studies (top 1%) in pharmacology and pharmacy research via InCites tool based on Web of Science Core Collection. VOSViewer software was used to visualize the results. The outcomes included development trends, countries, subject areas, research institutes, collaborative networks, and subject terms.

**Results:** We found 4,273 high-impact (top 1%) studies between 2011 and 2020 in the field of pharmacology and pharmacy. The number of studies increased from 366 in 2011 to 510 in 2020. These studies were mainly distributed in the following Web of Science subject categories: pharmacology and pharmacy (*n* = 4,188); neurosciences (*n* = 397); chemistry, multidisciplinary (*n* = 359); chemistry, medicinal (*n* = 314); microbiology (*n* = 301); biotechnology and applied microbiology (*n* = 280). These studies were cited in 646,855 studies from more than 100 Web of Science subject categories, and studies in pharmacology pharmacy accounted for the largest share of these citations. The top three countries that contributed the highest number of studies were the United States, United Kingdom, and China. The top three institutions that contributed the highest number of studies in the United States were the University of California System, the National Institutes of Health (NIH), and Harvard University. The top research collaborative circle was from universities in the United States. The top international collaborative circle was from universities from the United States, United Kingdom, Australia, and China. The subject-term analysis indicated that cancer was still the top disease, NF-κB was the top signaling pathway, and drug-delivery and nanoparticles were the top methods.

**Conclusion:** The high-impact studies in pharmacology and pharmacy research have grown over time. The United States, the United Kingdom, and China are the top countries that contributed the high-impact studies. Cancer is still the greatest challenge in the field of disease treatment. It calls for more international collaboration in pharmacology and pharmacy research, which will help discover novel drugs.

## Introduction

Pharmacology is the study of the sources, uses, and mechanisms of action of drugs (Reidenberg, 1991; Barnes et al., 2005). Pharmacy is the science or practice of preparing, formulating, and dispensing medicinal drugs (Allen, 2012; Shcherbakova and Desselle, 2020). In the Web of Science subject categories, “pharmacology and pharmacy” is a category that includes studies from both fields. With the rapid development of research methods, a substantial number of studies were published in this category (Krämer, 2008; Jones, 2014). However, the trends of studies in this category are still unclear.

Trends of a research field are usually reflected by high-impact studies (Hollenberg et al., 2020). Therefore, analyzing these high-impact studies could help us identify the research trends and foresee the potential developments in this research field (Chen et al., 2020; De Serrano, 2017; Zhu et al., 2020). High-impact studies are essential manifestations of first-class research findings (Becker et al., 2018). Hence, analysis of high-impact studies will provide an objective reflection of the hot research spots and the focus areas in such research (Oberbauer, 2020), which will serve as a valuable reference for the development of similar research and assessments of the academic impact of these studies (Glynn and Greenland, 2020; Janke et al., 2019). Because of the rapid growth of digital publishing, we can easily access studies in different research fields (Zhu et al., 2020). Previously, several studies were published in different fields, including nursing (Zhu et al., 2020), hand surgery (Eberlin et al., 2012), and other fields (Zhang et al., 2019; Zhang et al., 2020). A nursing study by Zhu et al. (2020) has analyzed the high-impact studies in nursing based on the InCites database and provided references for the nursing managers in the research field. The InCites database is a scientific research performance analysis tool based on more than 30 years of authoritative citation data of the Web of Science Core Collection (Bornmann and Leydesdorff, 2013; Dardas et al., 2019; Zhu et al., 2020), which is superior for analyzing high-impact studies than directly searching the Web of Science Core Collection (Zhu et al., 2020). Up to now, there is no comprehensive analysis of the high-impact studies in pharmacology and pharmacy.

This cross-sectional study analyzed the high-impact studies in pharmacology and pharmacy using the InCites tool based on the Web of Science Core Collection. The objectives include reporting high-impact studies’ dynamic trends, analyzing subject areas of high-impact studies, identifying top countries and institutions, exploring collaborative networks, and discovering hot research topics.

## Methods

### Study Design

A cross-sectional study was conducted.

### Inclusion and Exclusion Criteria

The inclusion criteria were as follows: 1) the research category should be “pharmacology and pharmacy” when using the InCites tool based on the Web of Science Core Collection (Zhu et al., 2020); 2) the article type should be article or review; 3) studies should be in the top 1%; 4) studies should be published in the past 10 years, from 2011 to 2020. The exclusion criteria were as follows: 1) article type was editorial, correction, or other types; 2) studies could not be found in the Web of Science Core Collection.

### Study Collection and Data Extraction

The initial retrieval date was April 19, 2021. We performed a search using InCites tool based on the Web of Science Core Collection. The following filters were used: research area = “Pharmacology and Pharmacy,” year = “2011–2020,” document type = “article or review,” study rank = “top 1%”. A total of 4,273 high-impact (top 1%) studies were included. The following data were downloaded from the InCites tool: author names, title, journal, organization, keywords, references, page numbers, Category Normalized Citation Impact (CNCI) (Zhu et al., 2020), and country. The 4,273 studies were then searched in the Web of Science Core Collection via the InCites tool. The details were extracted, including author names, title, journal, organization, keywords, references, page numbers, and subject categories.

### Statistical Analysis

Statistical analysis was performed using the following software: Excel and VOS Viewer. The outcomes included trends in the number and citation impact, subject areas, countries, institutions, collaborative networks, and subject terms. The citation impact was assessed using CNCI (Zhu et al., 2020), an indicator of the Web of Science for evaluating impact. A CNCI value of one indicates a citation performance on par with the world average, whereas CNCI values above and below one are considered above average and below average, respectively. A descriptive analysis was performed to assess the trends in the number and citation impact, subject areas, countries, and institutions. A visualization analysis was performed using VOS Viewer to evaluate the collaborative networks and subject terms.

## Results

### Trends in the Number and Citation Impact of High-Impact Studies in Pharmacology and Pharmacy Research

**FIGURE 1:**
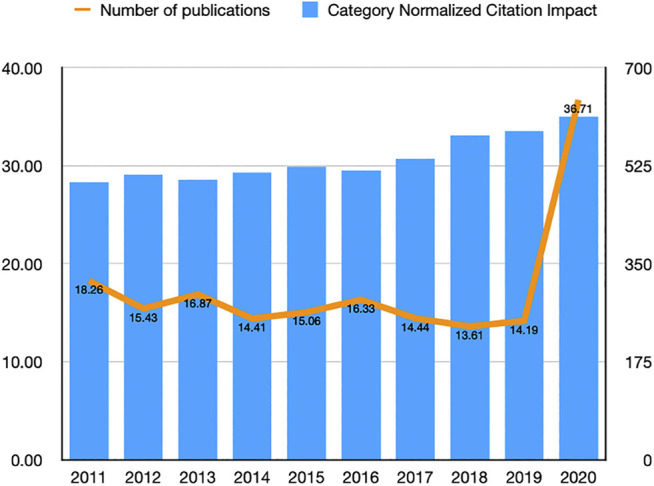
Number and citation impact of high-impact studies in pharmacology and pharmacy research from 2011 to 2020.

From 2011 to 2020, the number of high-impact pharmacology and pharmacy research studies increased from 495 to 613. The CNCI values decreased from 18.26 in 2011 to 14.19 in 2019 and dramatically increased in 2020 to 36.71 (Figure 1).

### Subject Categories of High-Impact Studies in Pharmacology and Pharmacy Research

The 4,273 high-impact studies were distributed to more than 100 Web of Science subject categories. Table 1 shows the 11 Web of Science subject categories with more than 100 studies each. After pharmacology and pharmacy (*n* = 4,188), neurosciences had the highest number of studies (*n* = 397), followed by chemistry, multidisciplinary (*n* = 359); chemistry, medicinal (*n* = 314); microbiology (*n* = 301); biotechnology and applied microbiology (*n* = 280). The 4,273 high-impact studies were cited by 646,855 studies, which were distributed in more than 100 Web of Science subject categories. The top 10 subject categories with the highest number of studies citing pharmacology and pharmacy research are shown in Table 2. Again, pharmacology and pharmacy accounted for the largest number of citations, with 124,423 citing studies. The citation impact of these high-impact studies extended to the categories of biochemistry and molecular biology (*n* = 72,550); chemistry, multidisciplinary (*n* = 53,574); neurosciences (*n* = 43,786); oncology (*n* = 36,180); chemistry, medicinal (*n* = 35,683); cell biology (*n* = 32,466); medicine, research, and experimental (*n* = 30,207); multidisciplinary sciences (*n* = 28,415); nanoscience and nanotechnology (*n* = 27,474).

**TABLE 1:** Eleven WOS subject categories that had more than 100 high-impact papers in pharmacology.

Ranking	Category	Number of studies
1	Pharmacology and pharmacy	4,188
2	Neurosciences	397
3	Chemistry, multidisciplinary	359
4	Chemistry. medicinal	314
5	Microbiology	301
6	Biotechnology and applied microbiology	280
7	Psychiatry	261
8	Toxicology	235
9	Infectious diseases	166
10	Clinical neurology	136
11	Medicine, research, and experimental	113

**TABLE 2:** Top 10 WOS subject categories in terms of the number of studies citing the high-impact studies in pharmacology and pharmacy research.

Ranking	Category	Number of studies
1	Pharmacology and pharmacy	124,423
2	Biochemistry and molecular biology	72,550
3	Chemistry, multidisciplinary	53,574
4	Neurosciences	43,786
5	Oncology	36,180
6	Chemistry, medicinal	35,683
7	Cell biology	32,466
8	Medicine, research, and experimental	30,207
9	Multidisciplinary sciences	28,415
10	Nanoscience nanotechnology	27,474

### Countries of Origin of the High-Impact Studies in Pharmacology and Pharmacy Research

**FIGURE 2:**
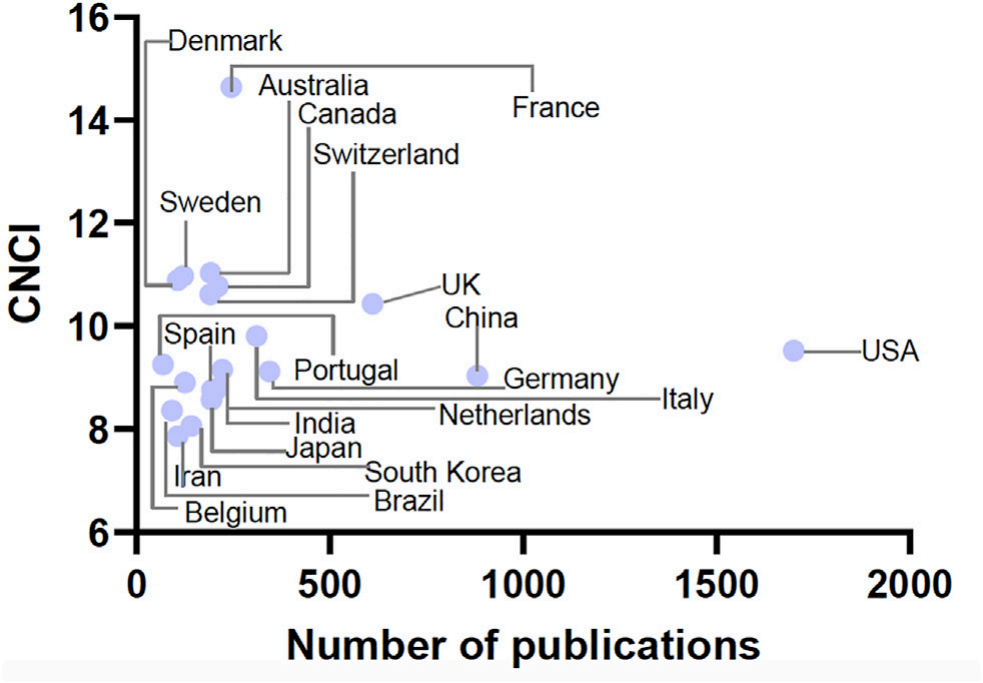
Number of high-impact papers in pharmacology and pharmacy research and CNCI value by country.

The countries that published the most high-impact studies in pharmacology and pharmacy research were the United States, United Kingdom, China, Germany, and Italy. In terms of CNCI values, the top five countries were France, Australia, Sweden, Denmark, and United Kingdom (Table 4 and Figure 2).

### Institutions of High-Impact Studies in Pharmacology and Pharmacy Research

**FIGURE 3:**
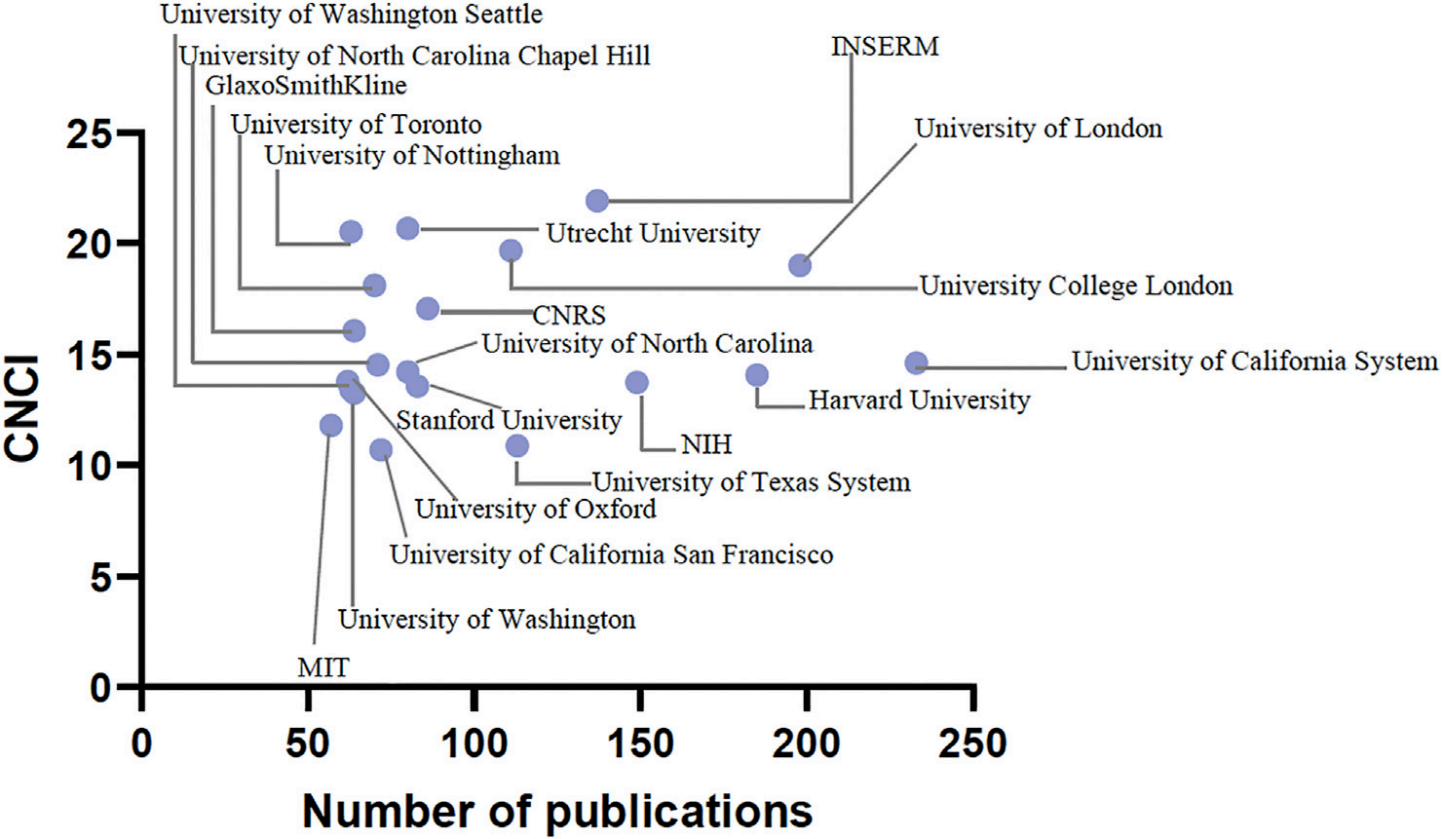
Number of high-impact studies in pharmacology and pharmacy research and CNCI values of the top 20 institutions.

The institutions that published the largest number of high-impact studies in pharmacology and pharmacy worldwide were the University of California System, National Institutes of Health (NIH), USA, Harvard University, University of London, and Institut National de la Sante et de la Recherche Medicale (INSERM). The total number of pharmacology and pharmacy research studies published by the top 20 originating institutions and their CNCI values is shown in Table 4 and Figure 3.

### Collaborative Networks of High-Impact Studies in Pharmacology and Pharmacy Research

**FIGURE 4:**
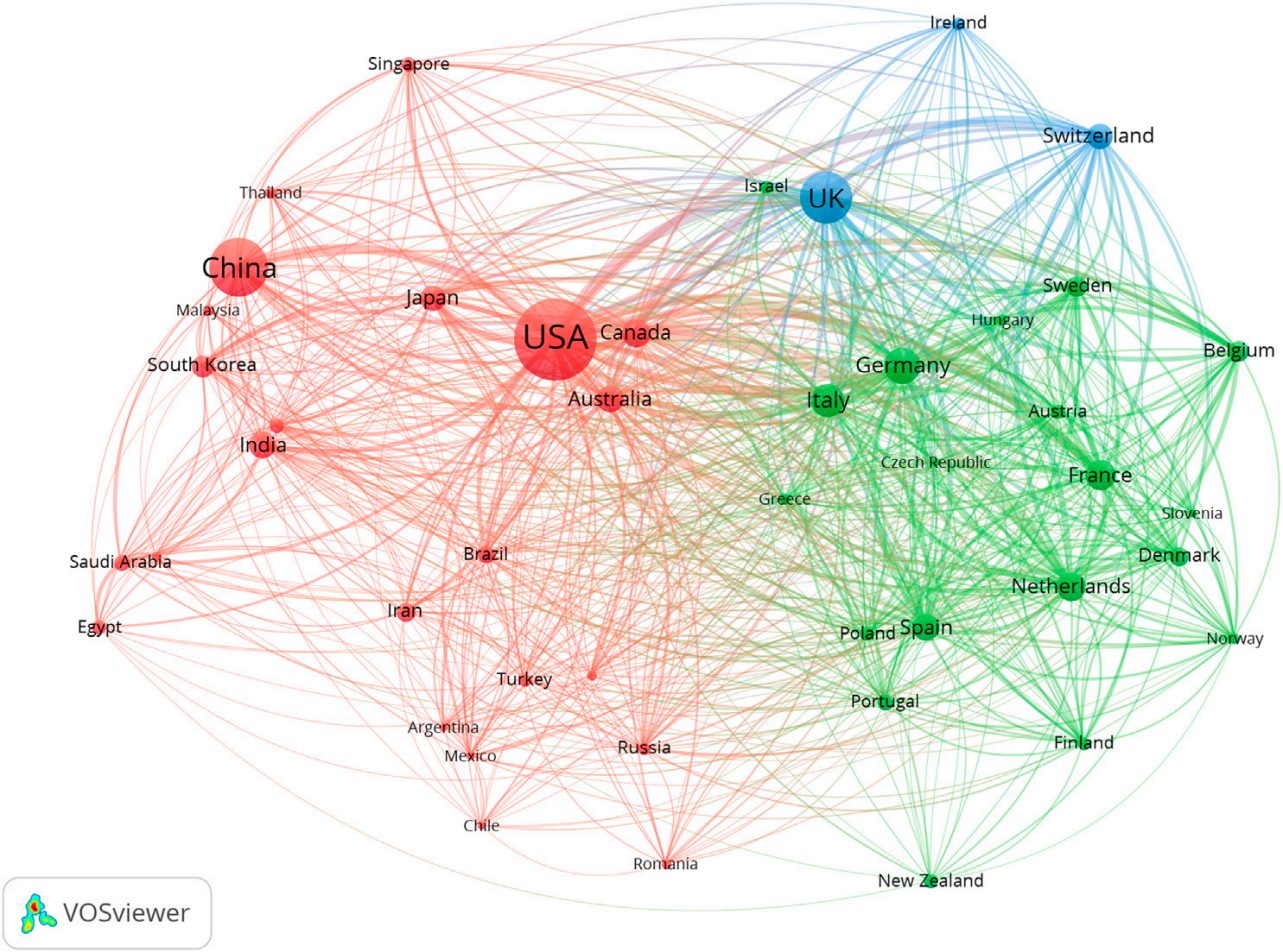
Map of collaborative networks among a country of high-impact papers in pharmacology and pharmacy research around the world.

**FIGURE 5:**
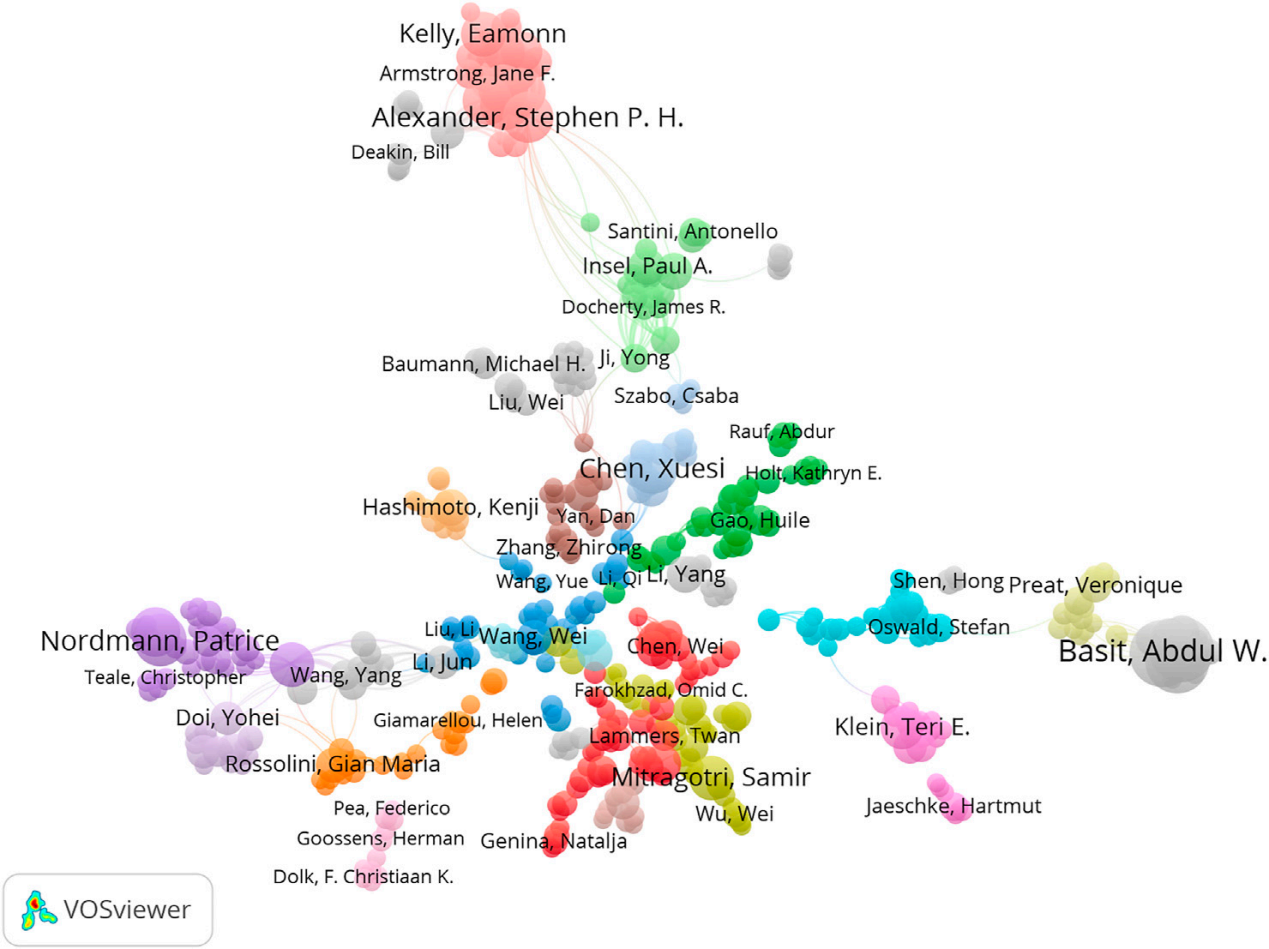
Map of collaborative networks among the authors of high-impact papers in pharmacology and pharmacy research around the world.

**FIGURE 6:**
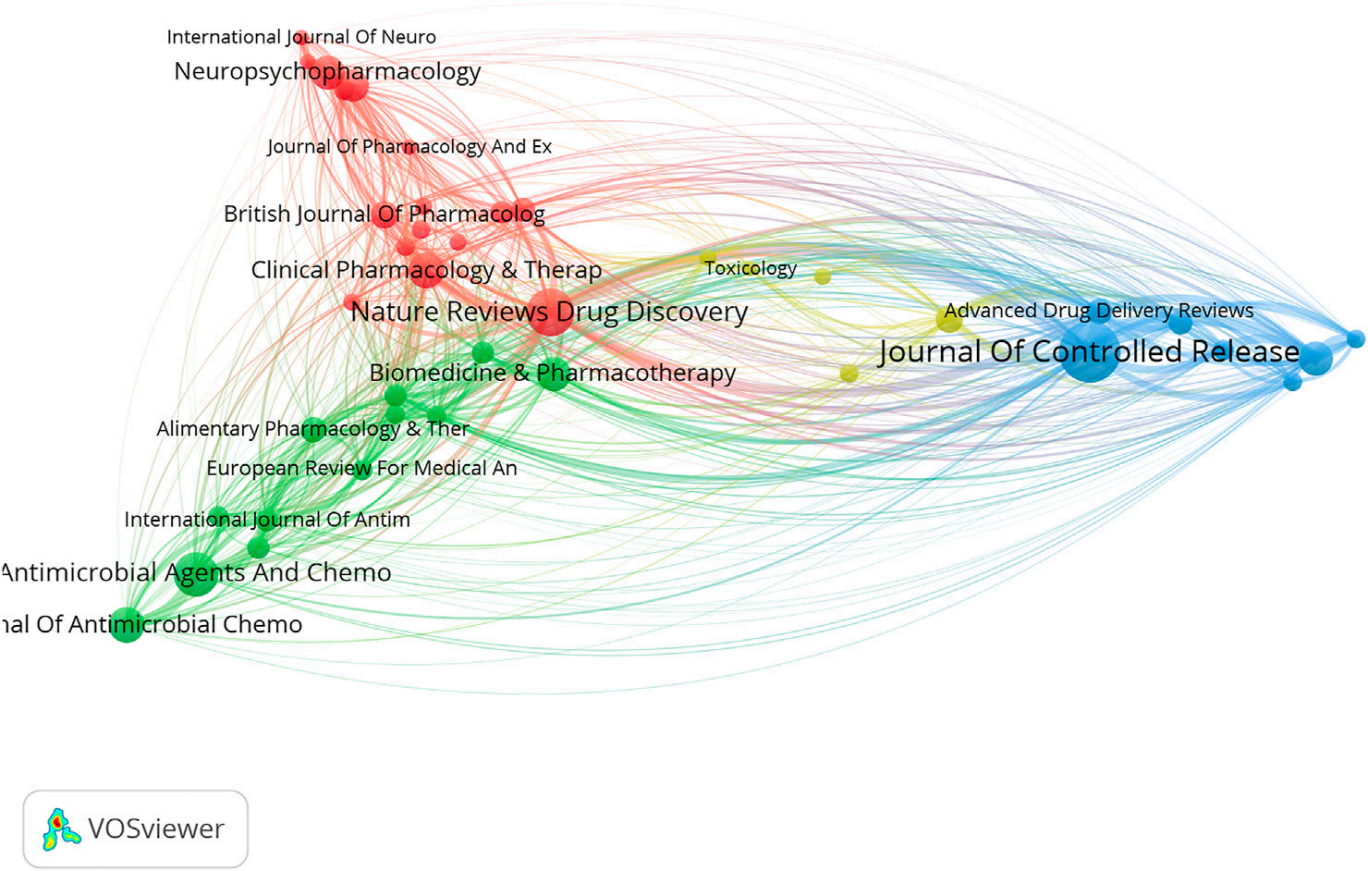
Map of collaborative networks among journals of high-impact papers in pharmacology and pharmacy research around the world.

**FIGURE 7:**
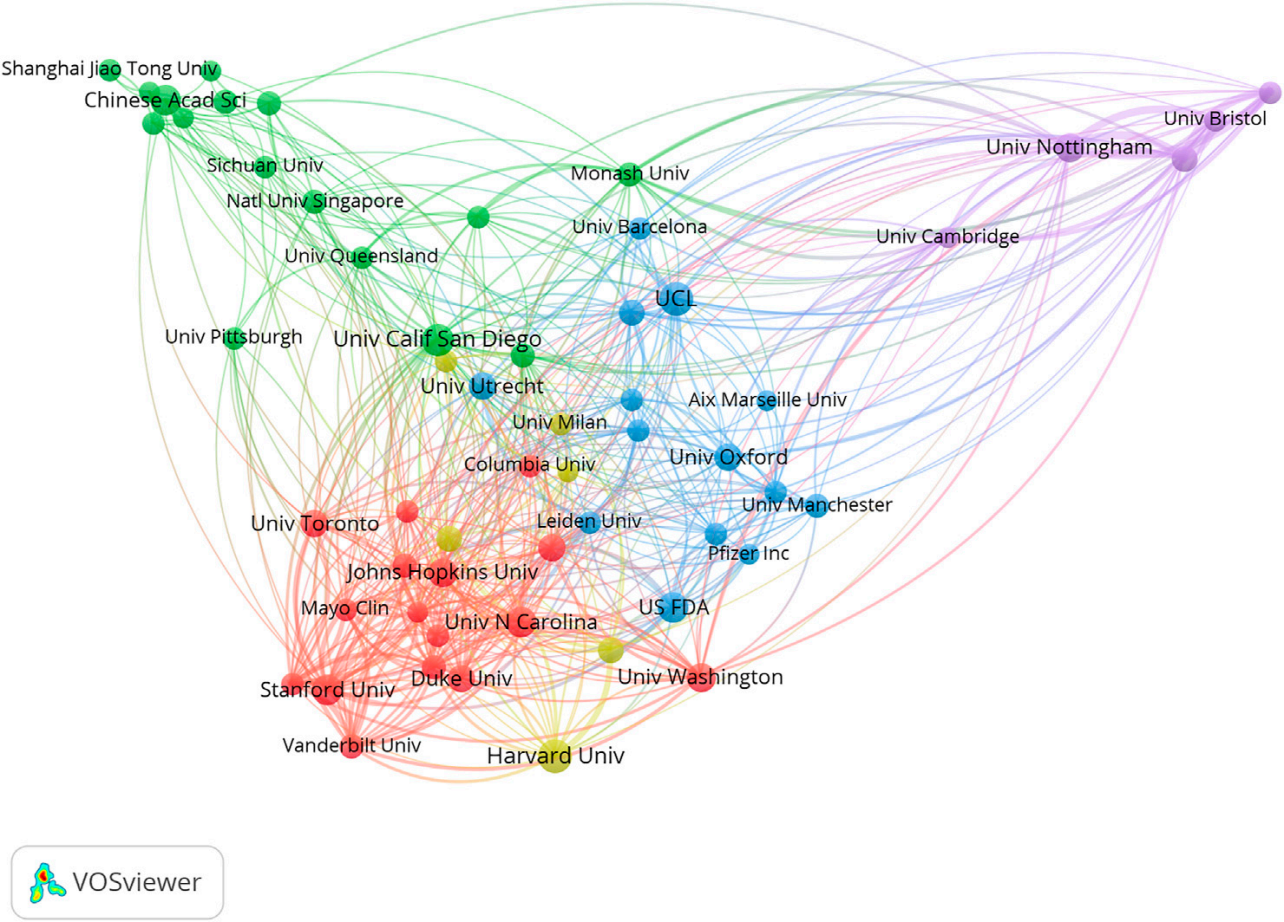
Map of collaborative networks among institutions of high-impact papers in pharmacology and pharmacy research around the world.

The collaborative networks were developed using VOS Viewer software. From the country level, four international collaborative circles were observed, among which the USA and China ranked first (red in Figure 4). Germany, France, and the Netherlands ranked second (green in Figure 4). Alexander Stephen ranked first at the author level, with 19 papers, 3,977 citations, and 178 links (Figure 5). The Journal of Controlled Release ranked first at the journal level, with 519 papers, 63,907 citations, and 964 links (Figure 6). At the institution level, there were four major collaborative circles (Figure 7). The largest node was the group of universities from the United States (red in the figure), including the University of Harvard, University of California System, University of Michigan, and John Hopkins University. The largest node of international collaborations was based on universities from England, Australia, the USA, and China.

### Heatmap of Subject Terms of High-Impact Studies in Pharmacology and Pharmacy Research

**FIGURE 8:**
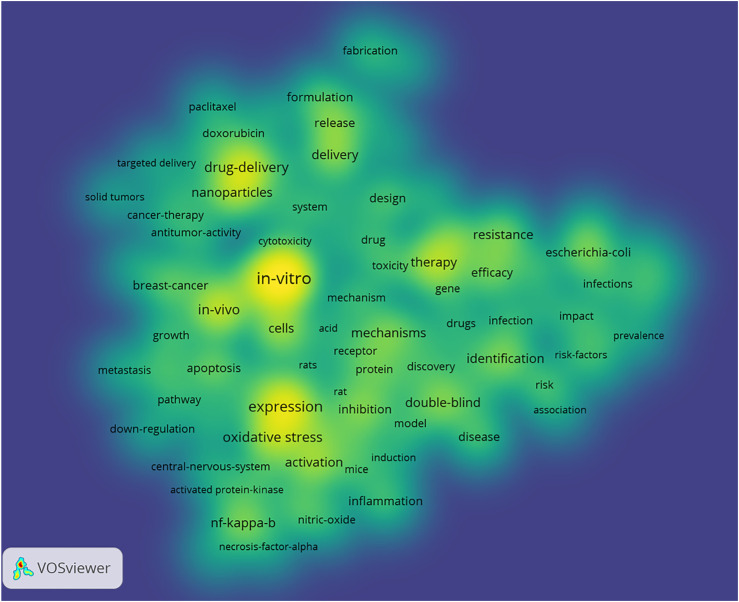
Heatmap of subject terms of high-impact studies in pharmacology and pharmacy research.

In Figure 8, the heatmap of subject terms indicated that “*in vitro*,” “expression”, “drug-delivery,” “cells,” “*in vivo*,” “oxidative stress,” “nanoparticles,” “therapy,” “activation,” “apoptosis,” “cancer,” “inhibition,” “NF-kappa-b,” “mechanisms,” “delivery resistance,” “efficacy,” “inflammation,” and “identification” were the core themes that received the most attention from researchers in pharmacology and pharmacy.

## Discussion

Analyzing high-impact studies helps understand the trends in a research field (Zhu et al., 2020); however, no such study has been performed in the field of pharmacology and pharmacy. Thus, we performed the current cross-sectional study. In this study, we enrolled 4,273 high-impact (top 1%) studies between 2011 and 2020 in pharmacology and pharmacy. The results indicated that the number of high-impact studies steadily increased from 2011 to 2020. The number of studies published in 2020 was the highest, which was quite different from a previous study on nursing (Zhu et al., 2020). In that study, studies in the latest years were less than those in the earlier years. Because citations were cumulated year by year, studies published in the latest years usually have a lower impact and lesser citations. In our analysis, studies in 2020 accounted for the highest yearly number of studies. This might be caused by the COVID-19 pandemic. The scientific field has paid more attention to the treatment of diseases, while the pharmacology and pharmacy field plays a vital role in treating diseases (Rizk et al., 2020; Rawat et al., 2021; Ghasemnejad-Berenji and Pashapour, 2021). Thus, studies got more attention and citations. Based on CNCI values, the citation impact of high-impact studies in pharmacology and pharmacy research was much higher than the global average of the citation impact of studies. Therefore, it might be inferred that academic interest in high-quality research in pharmacology and pharmacy is increasing over time.

The objective measures of citation analysis can be used to assess the value of published research findings (Zhu et al., 2020). The interactions between research areas could be measured by analyzing the variations in the subjects and citing studies. In our study, pharmacology and pharmacy studies were distributed in more than 100 WOS subject categories, including pharmacology and pharmacy; neurosciences; chemistry, multidisciplinary; chemistry, medicinal; microbiology. The high-impact studies were cited by research from more than 100 WOS subject categories, which suggests a wide range of influence (Oelgemöller and Hoffmann, 2016). Among the top subject categories, microbiology ranked fifth, which might be because of the COVID-19 pandemic (Rawat et al., 2021). From the top citing subject categories, neurosciences and oncology ranked in the top 5, suggesting that diseases from such fields are important. Our results showed that the high-impact studies have an influence on a wide range of subject categories. This interdisciplinary impact could promote more academic research and ensure its sustained development.

In our study, the countries that contributed the highest number of high-impact studies were the USA, UK, China, Germany, and Italy. Moreover, the highest CNCI values of high-impact studies were France, Australia, Sweden, Denmark, and the United Kingdom. The values of the seven countries were greater than 10, which means that these countries contribute most to the increase in CNCI value in 2020. The difference in countries between the number of studies and CNCI values could reveal the scientific impact of different countries in this field (Diaz et al., 2021; Xie et al., 2021).

Analyzing the institutions allows identifying the research institutions with the greatest attractiveness of talents (Zhu et al., 2020). Our results suggested that the University of California System had the most high-impact studies (*n* = 233). Although Utrecht University, INSERM, and the University of Nottingham did not publish the most high-impact studies, their CNCI values ranked in the top 3, and all were more than 20. Therefore, these universities or institutes might attract talents in such fields (Diaz et al., 2021; Xie et al., 2021). Notably, the CNCI value was relatively low for the United States. However, in Table 4, more than 50% of institutes from the USA were the top 20 institutes with relatively high CNCI values. The possible reason might be that, as shown in Table 3, the number of publications in the USA was 1700, and the total number of citations was 297,545. Table 4 shows that the number of USA publications of 11 institutions in the top 20 was 1,170, and the total number of citations was 244,868. The number of publications of the 11 institutions accounted for approximately 0.68 of the total USA publications, while the total number of citations accounted for 0.823 of the total number of USA citations. In other words, the number of studies published by other institutions from the USA accounted for only 0.32, and the number of citations only accounted for 0.177. This indicated that the number of citations of other institutions in the USA was relatively low. Therefore, the comprehensive calculation would lower the overall CNCI value, which would explain the relatively low CNCI values in Table 3 and the relatively high CNCI values in Table 4.

**TABLE 3:** Number of high-impact papers in pharmacology and pharmacy and CNCI value by country.

Rank of citation time	Country	Web of Science documents	Times cited	CNCI
1	United States	1,700	297,545	9.53
2	United Kingdom	610	100,292	10.44
3	China	882	75,638	9.05
4	Germany	344	50,635	9.13
5	France	245	41,317	14.64
6	Italy	311	41,080	9.82
7	Canada	211	39,815	10.78
8	Netherlands	222	34,963	9.16
9	Switzerland	190	34,012	10.62
10	Australia	192	31,072	11.04
11	Japan	194	29,757	8.58
12	India	211	28,101	8.81
13	Spain	195	25,146	8.77
14	Sweden	121	23,138	10.98
15	Belgium	125	22,160	8.91
16	Denmark	106	20,855	10.90
17	South Korea	142	16,643	8.07
18	Portugal	68	10,827	9.26
19	Brazil	92	10,750	8.37
20	Iran	106	10,109	7.87

**TABLE 4:** Top 20 institutions of high-impact papers in pharmacology and pharmacy research.

Rank	Institution	Web of Science documents	Times cited	CNCI
1	University of California System	233	39,918	14.62
2	NIH	149	38,684	13.73
3	Harvard University	185	37,733	14.07
4	University of London	198	30,007	19.00
5	INSERM	137	24,528	21.92
6	University of Texas System	113	24,518	10.89
7	University of Washington	64	16,249	13.27
8	University of Washington Seattle	63	16,123	13.40
9	University of North Carolina	80	15,612	14.21
10	MIT	57	14,792	11.80
11	Utrecht University	80	14,670	20.68
12	University of Toronto	70	14,539	18.11
13	University College London	111	14,066	19.68
14	University of North Carolina Chapel Hill	71	13,926	14.54
15	University of Oxford	62	13,855	13.78
16	Stanford University	83	13,807	13.58
17	University of California San Francisco	72	13,506	10.69
18	CNRS	86	13,449	17.07
19	University of Nottingham	63	13,355	20.53
20	GlaxoSmithKline	64	12,289	16.07

In the collaborative network analysis, the results suggested that the largest research collaborative node was the group of universities in the United States. Hence, these universities collectively possessed the most significant influence and research capacity, and were the core research institutions. In addition, the geographical distribution of these institutions indicated the same region or neighboring regions, which is consistent with previous studies in the nursing field (Zhu et al., 2020). The largest node of international collaborations was based on universities from England, Australia, the USA, and China, suggesting that international collaborations were critical in the top studies of such fields, which was consistent with the results of many studies (Chong et al., 2021; Li et al., 2021; Zhang S. et al., 2021). This indicates that the academic capacity of an institution is positively related to its cooperative relationship (Chong et al., 2021; Li et al., 2021; Zhang S. et al., 2021).

In the heatmap, we found that *in vitro*, expression, drug-delivery, cells, and *in vivo* ranked in the top five core themes in this field, suggesting that these were the essential concepts in this field. In addition, cancer had the most significant term frequency in the literature because it is still the greatest challenge in terms of treatment (Vasan et al., 2019). Cancer treatment is the most prominent topic for high-impact studies in this field (Jin et al., 2021; Li et al., 2020; Liu et al., 2020). We also found that oxidative stress and inflammation were the core themes, which predicted targets of drug therapy or drug discovery in the future (Reuter et al., 2010). Notably, the NF-κB pathway was among the core themes, suggesting that the signaling pathway is still a major pharmacology research topic (Eluard et al., 2020).

There were several limitations of this study. First, the InCites tool based on the Web of Science Core Collection was used to analyze the data. Data analysis from a second database might produce different results (Zhang S. et al., 2021). Second, only articles and reviews were included. The results might be biased because of enrolled types of studies, especially editorials from top scientists. Third, further analysis of the top 100 cited papers, authors, or institutions might provide meaningful results (Zhang et al., 2020; Zhang S. et al., 2021; Zhang Y. et al., 2021). Fourth, as there was no clear definition of high-impact papers, we included the top 1% studies. Other analytical criteria might be useful, such as analyzing the top 10% papers or the top 100 cited papers. Fifth, the data will change with time. Therefore, the conclusions should be updated in the future.

Despite the limitations mentioned above, this study provides general trends in high-impact studies in pharmacology and pharmacy research published from 2011 to 2020. Our study suggests that high-impact studies have grown over time. The United States, United Kingdom, and China are the top countries that contributed the high-impact studies. Cancer is still the leading challenge in the field of disease treatment. It calls for more international collaboration in this field, which will help in the discovery of drugs.

## Data Availability

The raw data supporting the conclusions of this article will be made available by the authors, without undue reservation.
